# Evaluation of changes in the expression of Wnt/β-catenin target genes in mouse reproductive tissues during estrous cycle: An experimental study

**Published:** 2018-02

**Authors:** Saeed Zavareh, Zahara Gholizadeh, Taghi Lashkarbolouki

**Affiliations:** 1 *School of Biology, Damghan University, Damghan, Iran. *; 2 *Institute of Biological Sciences, Damghan University, Damghan, Iran. *

**Keywords:** Wnt signaling pathway, Beta catenin, Estrous cycle, Mice

## Abstract

**Background:**

The Wingless-type (Wnt)/β-catenin signaling pathway controls cell homeostasis. Reproductive tissues are dynamic in response to steroidal hormone changes. Steroidal hormones are known to control the Wnt/β-catenin pathway, but their role in reproductive tissues remains unknown.

**Objective:**

The present study aims to investigate the expression patterns of Wnt/β-catenin target genes in mouse reproductive tissues during the normal estrous cycle.

**Materials and Methods:**

In this experimental study, 16 adult NMRI mice were grouped as proestrus, estrus, metestrus, and diestrus according to vaginal smear and histological evaluation of uterine and ovarian tissues. Uterine horns and ovarian tissues were collected. Reverse transcription quantitative polymerase chain reaction was performed to evaluate the expression of Wnt/β-catenin target genes (*Myc2, Ppard, Id2, Birc5, *and* Ascl2*) at different stages of the estrous cycle.

**Results:**

The expression levels of *Id2*, *Ascl2*, and *Pprd* in uterine tissue were significantly higher at the proestrus phase than at the other stages. Meanwhile, *Birc5* expression in uterine tissue was significantly higher at the metestrus stage than at the other stages. Furthermore, *Myc2* expression was significantly higher at the diestrus stage than at the estrus and metestrus stages. In the ovarian tissue, the highest expression of *Id2*, *Ascl2*, and *Birc5* was detected at the proestrus stage, whereas the highest expression of *Myc2* and *Ppard* was observed at the estrus stage.

**Conclusion:**

This study showed that Wnt/β-catenin target genes profiles are different among estrous cycle. It seems that different hormonal profiles during estrous cycles play a key role in the expression pattern of Wnt/β-catenin target genes in ovarian and uterine tissue.

## Introduction

The reproductive system is unique for its distinguished cellular turnover during each reproductive cycle (i.e., menstrual and estrous cycles). Cellular changes in the uterine involve regeneration, differentiation, and shedding of endometrium tissue during the menstrual cycle or growth and apoptosis during the estrus cycle. The immature follicles embedded in ovarian tissue pass finally through different stages of development and ovulate. Cellular remodeling of uterine tissue is attributed to steroidal hormones in the ovary, and ovarian tissue is affected by the steroidal hormones it secretes (1, 2). 

Studies on reproductive biology have recently focused on Wingless-type (Wnt) signaling. Steroidal hormones affect the Wnt signaling pathways in reproductive organs and have been discussed as a target for endocrine disorders. Wnt signaling also regulates many vital aspects of developmental and physiological processes, such as cell growth and proliferation, generation of cell polarity and differentiation, homeostasis, apoptosis, and cell-cell interactions during embryogenesis (3-6).

The Wnt signaling system is activated via two pathways: canonical (β-catenin-dependent) and non-canonical (β-catenin-independent) which in turn control the expression of its target genes. Canonical Wnt signaling affects specific target genes via members of the transcription factor T cell/lymphoid enhancer binding transcription factor (Lef/Tcf) family (6). The expression of target genes of β-catenin pathways regulates cellular biology and tissue homeostasis. For example, the *Myc2*, Ccnd1, and *Ppard* genes regulate cell proliferation, whereas *Ascl2* and Vegf regulate stem cell fate determination and angiogenesis, respectively. Furthermore, the *Id2*, Itf2, and Enc1 genes have been implicated in regulating cell differentiation (5-7). 

Wnt signaling plays a key role in the differentiation of uterine endometrium during the menstrual or estrous cycle (3-5). In this regard, the expression levels of Wnt-2, Wnt-3, Wnt-4, Wnt-5a, Wnt-7a, and Wnt-7 were detected in the normal uterine endometrium during the menstrual cycle (8, 9). It was shown that estrogen activates canonical Wnt signaling, which is essential for the proliferation of epithelial cells but exerts no effect on uterine stromal cells (3-6). However, the regulatory effects of steroidal hormones on Wnt signal target genes remain unidentified. The interaction of β-catenin signals and estrogenic receptors changes DNA transcription. Nucleotide sequences were distinguished by both Tcf and estrogen receptors. In addition, Tcf and estrogen receptors in the presence or absence of DNA could interact and form a complex as an antagonist or stimulating factor depending on the target gene promoter activity, which in turn sets up a dialogue to induce late estrogenic growth response (3-5, 8, 9). 

Four phase including proestrus, estrus, metestrus, and diestrus have organized the estrous cycle: At each phase of the estrous cycle, specific changes in the uterus and ovaries, such as cellular proliferation, apoptosis, and differentiation, are affected by steroidal hormones (10). 

To the best of our knowledge, the expression pattern of β-catenin target genes during the estrous cycle has not yet been reported. Hence, in the present study, the expression pattern of β-catenin target genes examined in ovarian and uterine tissues during different phase of normal estrous cycle.

## Materials and methods

**Animals**

In this experimental study, 6-8-wk-old virgin female National Medical Research Institute mice (n=16) were purchazed from the Pasteur Institute of Iran. Animals were acclimated for at least 7 days before any experimental manipulations. The animals were cared and housed in standard cages in a room with controlled temperature (22-24^o^C) and humidity (30-70%), as well as 12 hr light/dark cycle, lights on at 6:00 a.m. At least four animals were examined at each experiment point. 

**Histological analysis of estrous**

The stages of the estrous cycle were determined by first evaluating the vaginal orifice, confirming the phases of the estrous cycle by vaginal cytology, and finally conducting a histology of uterine and ovarian tissues as previously described (10). In brief, every morning, each animal was examined for vaginal appearance. The criteria of visual observation of the vaginal orifice to evaluate the stage of the estrous cycle were in accordance with the defined norms by Bagheripour and colleagues (11). After visual observation of the vaginal orifice, the mice were sacrificed by cervical dislocation and their uterus and ovaries were removed and cytological evaluation of vaginal smears was performed. 

In brief, repeated pipetting and flushing of 10 µL of phosphate buffer saline (PBS; Sigma Aldrich, Cambridge, UK) in the vagina were conducted using a plastic pipette. Then, the content of vaginal flushing was loaded onto glass slides, fixed, and then stained with methanol and methylene blue (2%). The stained slides were assessed under a light microscope (Nikon, Tokyo, Japan) with 10× and 40× objective lenses. Cells that were considered included nucleated epithelial cells, irregular cornified cells, and polymorphonuclear leukocytes. Previously described histological criteria were used to confirm the accuracy of the estrous cycle staging (10). 

In brief, the ovarian and uterine tissues were fixed in 4% paraformaldehyde (Merck, Darmstadt, Germany) for 12 hr at 4^o^C. Afterward, the tissues were dehydrated in increasing concentrations of ethanol series and then cleared in xylene (Merck, Darmstadt, Germany). Subsequently, they were embedded in paraffin wax, and 5 µm transverse microtome sections were stained with hematoxylin-eosin (H&E). The stained slides were assessed under a light microscope (Nikon, Tokyo, Japan) with 10× and 40× objective lenses.

**Reverse transcription quantitative polymerase chain reaction (RT-qPCR)**

The ovarian and uterine tissues at the different stages of the estrous cycle were excised and frozen in liquid nitrogen until examination. Total RNA was harvested with Trizol reagent (Qiagen) in accordance with the manufacturer’s instructions. Extracted RNA was dissolved in diethylpyrocarbonate-treated water and any DNA contamination was eliminated using RNAse-free DNAse. RNA quality was evaluated using the density ratio of 28S-18S rRNA bands. RT-qPCR was carried out on an ABI PRISM 7000 sequence detection system (Applied Biosystems, USA) using Fast SYBR® Green Master Mix Kit (Fermentase, USA). 

According to the program formulated in the pre-tests, relative change in gene expression was normalized with glyceraldehyde-3-phosphate dehydrogenase (*Gapdh*) and calculated using the ΔΔCt method. Detailed information of the primers used for amplification is provided in table I. Specificity of the PCR products was approved by analyzing of melting curve. In addition, owing to confirm the presence of a single right-sized PCR product, the samples were run on 2% agarose gels.

**Ethical consideration**

The Damghan University Institutional Animal Ethical Committee approved all experimental procedures (25: 2017). Free access to standard laboratory chow and water was provided.

**Statistical analysis**

All experiments were repeated at least four times. Statistical analysis was performed using SPSS (Statistical Package for the Social Sciences, version 19.0; SPSS Inc., Chicago, USA) and one-way analysis of variance (ANOVA) for real-time PCR results. Post-hoc Tukey’s HSD was used for multiple comparisons. Statistical significance was considered at p<0.05.

## Results

**Histological analysis of estrous**

**Figure 1:**
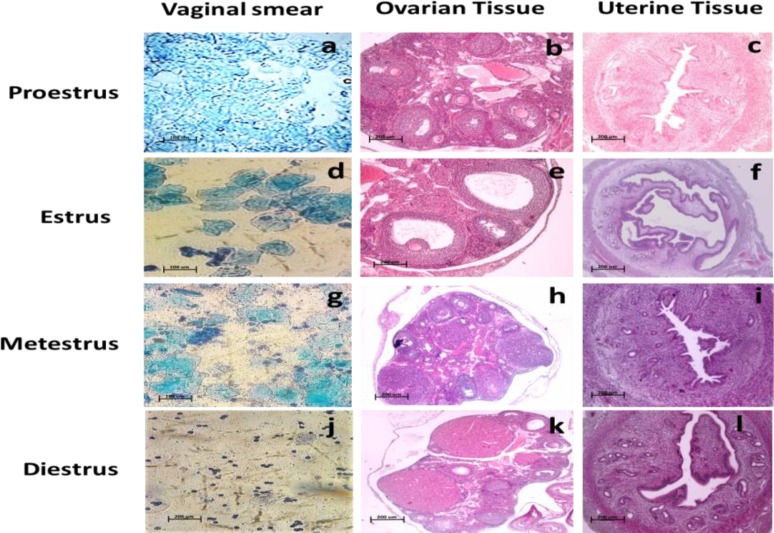
Stained vaginal smears and cross-section of uterine and ovarian tissue. (a) Proestrus vaginal smear. (b) Proestrus ovarian tissue. (c) Proestrus uterine tissue. (d) Estrus vaginal smear. (e) Estrus ovarian tissue. (f) Estrus uterine tissue. (g) Metestrus vaginal smear. (h) Metestrus ovarian tissue. (i) Metestrus uterine tissue. (j) Diestrus vaginal smear. (k) Diestrus ovarian tissue. (l) Diestrus uterine tissue. Scale bar= 200 µm

The cytological appearance of mouse vaginal flushing and the histological properties of the uterine and ovarian tissues that define the four stages of the estrous cycle are shown in figure 1. Vaginal smear of the proestrus phase showed the dominant presence of nucleated cells and some cornified epithelial cells (Figure 1 a). While, in the estrus stage, cornified epithelial cells were dominant (figure 1d). An equal proportion of cornified epithelial cells, polymorphonuclear leukocytes, and some nucleated epithelial cells were characteristic of the metestrus vaginal smear (Figure1g). In addition, vaginal smear of diestrus phase showed mostly polymorphonuclear leukocytes and some epithelial cells (Figure 1j). The histological features of the uterus in the proestrus stage (Figure 1b) were characterized by the presence of leukocyte infiltration, and luminal dilation. Meanwhile, the ovarian tissue in the proestrus phase showed several tertiary follicles and degenerated corpus luteum (Figure 1c). 

At the estrus stage, uterine histological features were large and tall columnar cells of the endometrial layerlumen dilation and infiltrated leukocytes in the endometrial glands (Figure 1e). Several Graafian (preovulatory) follicles observed in the estrous ovarian section (Figure 1f). In the metestrus stage, mitotic figures increased and leukocyte infiltration decreased than that in the estrus stage (Figure 1h). In the ovarian section of the metestrus phase, corpus hemorrhagicum formed (Figure 1i). The uterus lumen in the diestrus stage (figure 1k) was small and slit-like with stromal edema. Moreover, large corpus luteum was detected in the ovarian tissue at the diestrus stage (Figure 1l).

**Reverse Transcription quantitative PCR**

**Figure 2:**
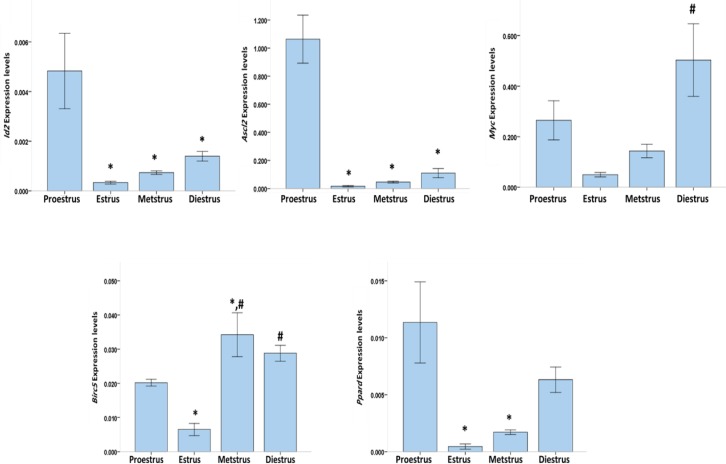
RT-qPCR results of the expression of *Myc2*,* Pprd*, *Id2*, *Birc5*, and* Ascl2* in the uterine tissue at the different stages of the estrous cycle. Error bars, ± 1 SD.

**Figure 3:**
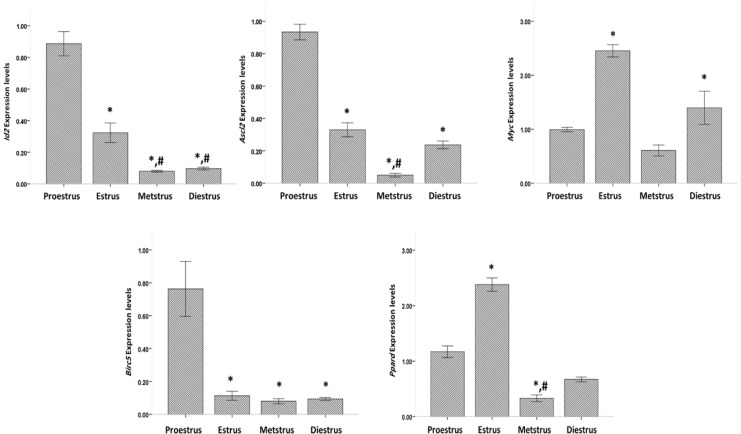
RT-qPCR results of the expression of *Myc2*,* Pprd*, *Id2*, *Birc5*, and* Ascl2* in the ovarian tissue at the different stages of the estrous cycle. Error bars, ± 1 SD.

Quantitative real-time PCR revealed the expression of *Myc2*, Pprd, *Id2*, *Birc5*, and *Ascl2* in the uterine and ovarian tissues during the estrous cycle (Figure 2, 3).

In the uterine tissue, comparison of the normalized Ct values (2-ΔΔct) showed that the mRNA expression levels of *Id2*, *Ascl2*, and Pprd were significantly higher at the proestrus stage than at the other stages of the estrous cycle. By contrast, no significant difference was found in the mRNA expression of *Id2*, *Ascl2*, and Pprd among the estrus, metestrus, and diestrus stages. Furthermore, the mRNA expression level of *Myc2* in the uterine tissue was significantly higher at the diestrus stage than at the estrus and metestrus stages. However, no significant difference in the mRNA expression of *Myc2* in the uterine tissue was found between the diestrus and proestrus stages as well as between the estrus and metestrus stages. The expression level of *Birc5* in the uterine tissue was significantly higher at the proestrus phase than at the estrus phase, whereas that of *Birc5* was significantly higher at the metestrus stage than at the estrus and proestrus stages (Figure 2).

The WNT target genes were expressed in the ovarian tissue at all stages of the estrous cycle. The expression levels of *Id2* and *Ascl2* were significantly higher at the proestrus stage than at the other stages of the estrous cycle. Meanwhile, the lowest expression of *Id2* and *Ascl2* was found at the metestrus stage in the ovarian tissue. Furthermore, the mRNA expression of Pprd in the ovarian tissue was significantly different among all stages of the estrous cycle. The mRNA expression level of Pprd was significantly higher at the estrus stage than at the other stages. Meanwhile, the expression level of Pprd was significantly higher at the proestrus stage than at the metestrus and diestrus stages. In addition, the lowest expression of Pprd was detected in the diestrus stage in the ovarian tissue. The mRNA expression level of *Myc2* in the ovarian tissue was significantly higher at the estrus stage than at the other stages of the estrous cycle. Moreover, no significant difference in the mRNA expression of *Myc2* in the ovarian tissue was found between the diestrus and proestrus stages. 

Furthermore, the *Myc2* expression in the uterine tissue revealed no significant differences between the proestrus and metestrus stages. By contrast, the expression level of *Myc2* in the uterine tissue was significantly higher at the diestrus phase than at the metestrus phase. In the ovarian tissue, the mRNA expression of *Birc5* was significantly higher at the proestrus stage than at the other stages of the estrous cycle. By contrast, no significant difference was observed in the mRNA expression of *Birc5* among the estrus, metestrus, and diestrus stages (Figure 3).

**Table I:** List of primers

**Primer**	**Sequence**	**Primer size**	**Product size**	**TM**
Myc2-F	5- GTTGGAAACCCCGCAGACAG-3	20	260	62.5
Myc2-R	5- CGACCGCAACATAGGATGGA-3	20		60.5
Ppard-F	5- CATGTCGCACAACGCTATCC-3	20	246	60.5
Ppard- R	5- TGCCACAGTGTCTCGATGTC-3	20		60.5
Id2-F	5- AGCATCCCCCAGAACAAGAAG-3	21	248	61.2
Id2-R	5- AATTCAGATGCCTGCAAGGAC-3	21		59.5
*Birc5*-F	5-TCCCGGCATGCTCTGC-3	16	208	55.9
*Birc5*-R	5-TCCGCCATTCGCTCTGG-3	17		57.3
Ascl2-F	5-CTCTTGGGGCTTAAGGGCTG-3	20	253	62.5
Ascl2-R	5-CCGTACCAGTCAAGGTGTGC-3	20		62.5
Gapdh-F	5-TGACATCAAGAAGGTGGTGAAGC-3	25	203	60.6
Gapdh-R	5-CCCTGTTGCTGTAGCCGTATTC-3	22		62.1

*Myc2*: Myelocytomatosis oncogene

*Ppard*: Peroxisome proliferator activator receptor delta

*Id2*: Inhibitor of DNA binding 2

*Birc5*: Baculoviral IAP repeat-containing 5

F: Forward

*Gapdh*: Glyceraldehyde-3-phosphate dehydrogenase

*Ascl2*: Achaete-scute family bHLH transcription factor 2

R: Reverse

*: Represent the significant difference with Proestrus (p<0.05). **#**: Represent the significant difference with Estrus (p<0.05).

**#**: Represent the significant difference with Estrus (p<0.05).

*: Represent the significant difference with Proestrus (p<0.05).

**#**: Represent the significant difference with Estrus (p<0.05).

## Discussion

The results of the present study showed that the expression pattern of target genes in the Wnt/β-catenin pathway is different in the uterine and ovarian tissue at the different stages of the estrous cycle.

The uterus is the primary target of steroidal hormones produced from the ovary during reproductive cycles. The ovaries are also affected by the steroidal hormones they secrete (1). Members of the Wnt family play important roles in the early stages of embryonic development, as well as in adult homeostasis. It is noteworthy that Wnt family members are affected by steroidal hormones (3, 4). Estrogen induces the proliferation of uterine epithelial cells and is essential for the preservation of secretory activity, cellular differentiation, and normal morphology of the epithelial cells of the uterus (1). 

The general consensus is that the estrogen in the uterus exerts its effects through the activation of nuclear receptors (12). In addition to the classic pathway, estrogen activates the conventional Wnt signaling in the uterus through an independent method (13). However, the relation of the estrogen biphasic response to the molecular events in the uterus remains unclear to date. The canonical Wnt signaling is immediately activated in response to estrogen, which is important for estrogen-dependent growth in the delayed phase. In addition, Wnt signaling connects the fast and delayed phases (9, 14). Changes in the estrous cycle are well simulated under anatomical and physiological conditions. During the pre-ovulatory period (proestrus), ovarian follicles secrete E2 and reach a peak at the estrus phase, when ovulation occurs. Then, progesterone (P4) levels rise during metestrus and reach a peak at the diestrus phase, followed by decrement and increment of P4 and E2, respectively, and start another cycle (15).

In the present study, the expression of some target genes of the Wnt/β-catenin pathway, including *Myc2*, *Ppard*, *Id2*, *Birc5*, and *Ascl2*, was investigated at the different stages of the estrus cycle in the uterus and ovaries. The highest expression of *Id2*, *Ascl2*, and *Ppard* in the mouse uterus was observed at the proestrus stage. At this stage, the level of estrogen increases, whereas that of P4 decreases, and the expression of the downstream gene expression increases, given that estrogen activates the Wnt signaling pathway (3). 

In addition, the aforementioned genes in the estrus stage had the lowest expression levels. This result indicates that the estrogen levels at this stage decrease while the P4 levels increase; therefore, the induced estrogen effect is reduced (3). *Ppard* regulates the transcription of genes involved in metabolic pathways, specifically in the transmission of fatty acids (16). Thus, *Ppard* is involved in the development of metabolic disorders in the body (16). In the present study, the expression of this gene increased at the proestrus stage, which can be expected considering that increased metabolic activity occurs in the estrus phase. Moreover, the expression of the *Ascl2* gene at the proestrus stage significantly differed from that at the other stages. This pattern of expression is similar to the expression of this gene in implantation and pregnancy, i.e., the expression of this gene is high at the early stages of pregnancy and then decreases (17). In addition, the expression pattern of the *Id2* gene varies with sex (18). 

Thus, the expression of this gene is influenced by the profile of individual steroidal hormones (18). Regarding the effect of steroidal hormones on the Wnt pathway, the gene expression varies during the different phases of the estrus cycle. In addition, the results of the present study showed that *Myc2* was significantly increased in the proestrus stage compared with the estrus, but its expression rate at the diestrus stage was significantly higher than those at the proestrus and estrus stages. The expression of the *Myc2* gene is low during cellular differentiation (19) The reduction of its expression at the estrus and proestrus stages is possibly due to the change in the status of the uterus. *Myc2* is a nuclear factor that contributes to the regulation of a wide range of biological processes, including division control, apoptosis, cell growth, angiogenesis, and differentiation (19). 

These features of *Myc2* prepare the uterine endometrium for the proliferation phase of the reproductive cycle. The results of previous experiments indicated that *Myc2* plays an unexpected role in controlling the self-renewal of stem cells (20). Evidence suggests that *Myc2* affects the differentiation of stem cells by a mechanism independent of its effect on division and survival (20). The presence of stem cells in the uterus was confirmed earlier.

*Birc5* is the only apoptotic suppressor protein that acts as a mitotic regulator (21). This is consistent with the results of the present study, which shows that *Birc5* expression increased at the metestrus and diestrus stages, which are both secretion phases, and the uterine endometrium thickened. Inhibition of *Birc5* increases cell death through apoptosis. Downregulation of *BIRC5* expression at the proestrus stage is relatively a reflection of this issue, as in the late estrus period, necrosis and thinning of the uterine endometrium begin (16).

In addition, changes in the expression of target genes of the Wnt/β -catenin pathway were observed in the ovarian tissue at the different stages of the estrous cycle. Wnt signaling pathways are involved in the regulation of ovarian function and, consequently, contribute to normal ovarian function and fertility. The widespread physiological involvement of Wnt signaling in the adult ovary remains unclear. Wnt family gene expression of rodent ovaries is affected by hormones. In this regard, administration of human chorionic gonadotropin elevated the Wnt4 expression in rat granulosa cells (22). 

Previous investigation showed that the Wnt pathway and downstream components influence the developing follicle and corpus luteum of rats, mice, humans, and cattle (22-26). Steroid production followed by Follicle stimulating hormone (FSH) secretion in the granulosa cells of antral follicles regulates the β-catenin signaling pathway because an increase in β-catenin protein is accompanied by high intrafollicular estradiol concentrations (25). In agreement with the role of β-catenin in ovarian follicle steroidogenesis, FSH can stimulate the β-catenin pathway and downstream target genes (25, 27, 28). Therefore, the β-catenin pathways support FSH-mediated activities in ovarian follicular cells (29). 

The β-catenin pathway plays a role in P4 production from the bovine corpus luteum after luteinizing hormone stimulation, However, β-catenin alone is insufficient to modulate steroidogenesis, and gonadotropins are necessary to maximize the effect of β-catenin on steroidogenesis in follicle cells (28). Thus, β-catenin contributes to the gonadotropin induction of ovarian steroidogenesis (27).

## Conclusion

In conclusion, the expression pattern of target genes in the Wnt/β-catenin pathway is different in the uterine and ovarian tissue at the different stages of the estrous cycle. This result indicates that expression of the target genes of the Wnt/β-catenin route is according to changes of the hormonal profile at the different stages of the estrous cycle.

## Note

***This article extracted from M.Sc. thesis. (Zahra Gholizadeh)***

## References

[1] Wood GA, Fata JE, Watson KL, Khokha R (2007). Circulating hormones and estrous stage predict cellular and stromal remodeling in murine uterus. Reproduction.

[2] Heffner LJ, Schust DJ (2010). The reproductive system at a glance.

[3] Hou X, Tan Y, Li M, Dey SK, Das SK (2004). Canonical Wnt signaling is critical to estrogen-mediated uterine growth. Mol Endocrinol.

[4] Tulac S, Nayak NR, Kao LC, Van Waes M, Huang J, Lobo S (2003). Identification, characterization, and regulation of the canonical Wnt signaling pathway in human endometrium. J Clin Endocrinol Metab.

[5] Katayama S, Ashizawa K, Fukuhara T, Hiroyasu M, Tsuzuki Y, Tatemoto H (2006). Differential expression patterns of Wnt and β-catenin/TCF target genes in the uterus of immature female rats exposed to 17α-ethynyl estradiol. Toxicol Sci.

[6] Cadigan KM, Nusse R (1997). Wnt signaling: a common theme in animal development. Genes Dev.

[7] Herbst A, Jurinovic V, Krebs S, Thieme SE, Blum H, Göke B (2014). Comprehensive analysis of β-catenin target genes in colorectal carcinoma cell lines with deregulated Wnt/β-catenin signaling. BMC Genomics.

[8] Bui TD, Zhang L, Rees MC, Bicknell R, Harris AL (1997). Expression and hormone regulation of Wnt2, 3, 4, 5a, 7a, 7b and 10b in normal human endometrium and endometrial carcinoma. Br J Cancer.

[9] Miller C, Sassoon DA (1998). Wnt-7a maintains appropriate uterine patterning during the development of the mouse female reproductive tract. Development.

[10] Choobineh K, Zavareh S, Salehnia M, Ghorbanian MT, Paylakhi SH (2016). Expression of pluripotent stem cell markers in mouse uterine tissue during estrous cycle. Vet Res Forum.

[11] Bagheripour N, Zavareh S, Ghorbanian MT, Paylakhi SH, Mohebbi SR (2017). Changes in the expression of OCT4 in mouse ovary during estrous cycle. Vet Res Forum.

[12] Couse JF, Korach KS (1999). Estrogen receptor null mice: what have we learned and where will they lead us?. Endocr Rev.

[13] Mericskay M, Kitajewski J, Sassoon D (2004). Wnt5a is required for proper epithelial-mesenchymal interactions in the uterus. Development.

[14] Miller C, Pavlova A, Sassoon DA (1998). Differential expression patterns of Wnt genes in the murine female reproductive tract during development and the estrous cycle. Mech Dev.

[15] Wood GA, Fata JE, Watson KL, Khokha R (2007). Circulating hormones and estrous stage predict cellular and stromal remodeling in murine uterus. Reproduction.

[16] O'Connor DS, Wall NR, Porter AC, Altieri DC (2002). A p34 (cdc2) survival checkpoint in cancer. Cancer Cell.

[17] Peddada S, Yasui DH, LaSalle JM (2006). Inhibitors of differentiation (ID1, ID2, ID3 and ID4) genes are neuronal targets of MeCP2 that are elevated in Rett syndrome. Hum Mol Genet.

[18] Park HJ, Hong M, Bronson RT, Israel MA, Frankel WN, Yun K (2013). Elevated Id2 expression results in precocious neural stem cell depletion and abnormal brain development. Stem Cells.

[19] Schuhmacher M, Staege MS, Pajic A, Polack A, Weidle UH, Bornkamm GW (1999). Control of cell growth by c-Myc in the absence of cell division. Curr Biol.

[20] Wilson A, Murphy MJ, Oskarsson T, Kaloulis K, Bettess MD, Oser GM (2004). c-Myc controls the balance between hematopoietic stem cell self-renewal and differentiation. Genes Dev.

[21] Li F, Ambrosini G, Chu EY, Plescia J, Tognin S, Marchisio PC (1998). Control of apoptosis and mitotic spindle checkpoint by survivin. Nature.

[22] Hsieh M, Johnson MA, Greenberg NM, Richards JS (2002). Regulated expression of Wnts and Frizzleds at specific stages of follicular development in the rodent ovary. Endocrinology.

[23] Harwood BN, Cross SK, Radford EE, Haac BE, De Vries WN (2008). Members of the WNT signaling pathways are widely expressed in mouse ovaries, oocytes, and cleavage stage embryos. Dev Dyn.

[24] Wang Y, Hanifi-Moghaddam P, Hanekamp EE, Kloosterboer HJ, Franken P, Veldscholte J (2009). Progesterone inhibition of Wnt/β-catenin signaling in normal endometrium and endometrial cancer. Clin Cancer Res.

[25] Castañon BI, Stapp AD, Gifford CA, Spicer LJ, Hallford DM, Hernandez Gifford JA (2012). Follicle-stimulating hormone regulation of estradiol production: possible involvement of WNT2 and β-catenin in bovine granulosa cells. J Anim Sci.

[26] Gupta PS, Folger JK, Rajput SK, Lv L, Yao J, Ireland JJ (2014). Regulation and regulatory role of WNT signaling in potentiating FSH action during bovine dominant follicle selection. PloS One.

[27] Stapp AD, Gómez BI, Gifford CA, Hallford DM, Hernandez Gifford JA (2014). Canonical WNT signaling inhibits follicle stimulating hormone mediated steroidogenesis in primary cultures of rat granulosa cells. PloS One.

[28] Fan HY, O'connor A, Shitanaka M, Shimada M, Liu Z, Richards JS (2010). Beta-Catenin (CTNNB1) promotes preovulatory follicular development but represses LH-mediated ovulation and luteinization. Mol Endocrinol.

[29] Law NC, Weck J, Kyriss B, Nilson JH, Hunzicker-Dunn M (2013). Lhcgr expression in granulosa cells: roles for PKA-phosphorylated β-catenin, TCF3, and FOXO1. Mol Endocrinol.

